# Outcome of Chair-Side Dental Fear Treatment: Long-Term Follow-Up in Public Health Setting

**DOI:** 10.1155/2019/5825067

**Published:** 2019-06-04

**Authors:** T. Kankaala, T. Määttä, M. Tolvanen, S. Lahti, V. Anttonen

**Affiliations:** ^1^Department of Cariology, Endodontology and Paediatric Dentistry, Research Unit of Oral Health Sciences, University of Oulu, Oulu, Finland; ^2^Dental Teaching Unit, City of Oulu, Finland; ^3^Department of Community Dentistry, Institute of Dentistry, University of Turku, Turku, Finland; ^4^Center for Life Course Health Research, University of Oulu, Oulu, Finland; ^5^Medical Research Center, Oulu University Hospital and University of Oulu, Oulu, Finland

## Abstract

**Aim:**

Purpose of this practice and data-based study was to evaluate the outcome of dental fear treatment of patients referred to the Clinic for Fearful Dental Patients (CFDP) in the primary oral health care, City of Oulu, Finland, during period 2000–2005.

**Methods:**

A psychological approach including behavioral interventions and cognitive behavioral therapy (BT/CBT) was used for all participants combined with conscious sedation or dental general anesthesia (DGA), if needed. The outcome was considered successful if later dental visits were carried out without any notifications in the patient records of behavioral problems or sedation. Data collection was made in 2006; the average length of the observation period from the last visit in the CFPD to data collection was 2 y 3 m (SD 1 y 5 m). All information was available for 163 patients (mean age 8.9 y at referral). Study population was dominated by males (58.0%). Cause for referrals was mostly dental fear (81.0%) or lack of cooperation.

**Results:**

The success rate was 69.6% among females and 68.1% among males. Success seemed to be (*p*=0.053) higher for those treated in ≤12 years compared with the older ones. The participants, without need for dental general anesthesia (DGA) in the CFDP, had significantly a higher success rate (81.4%) compared with those who did (54.8%, *p* < 0.001). Use of conscious oral sedation (*p*=0.300) or N_2_O (*p*=0.585) was not associated with the future success.

**Conclusions:**

A chair-side approach seems successful in a primary health care setting for treating dental fear, especially in early childhood. Use of sedation seems not to improve the success rate.

## 1. Introduction

Every third Finnish child reports being afraid of at least something in dentistry [1]. The same is true also for adults among whom the prevalence has not shown any considerable decline over time [2]. Avoidance of dental treatment is the main consequence of dental fear; 41% of irregular use of oral health services is due to dental fear among Finnish adults [3]. Avoidance of dental treatment due to dental fear may lead to the deterioration of oral health and again further avoidance of oral health services which is described as a vicious circle [4, 5] sometimes resulting in need of dental treatment in general anesthesia (DGA) [6]. In Finland in 2010, 0.2% of the patients in public health in the Helsinki area received DGA [7]. However, DGA does not cure dental fear [8]. Dental fear may impair the oral health-related quality of life (OHRQoL) [9–12], and again, treatment of dental fear may lead to improvement in the OHRQoL [12].

The key element in dental fear treatment is enhancing patient's sense of trust and control by rapport and interactive communication [13]. Behavioral interventions (BT) are used sometimes combining them, if necessary with cognitive restructuring elements (CBT) [13, 14]. CBT/BT techniques comprise, i.e., cognitive restructuring, systematic desensitization, distraction, positive reinforcement, guided imaginary, muscle or breathing relaxation, tell-show-do, and rest breaks. In their review article, Wide Boman et al. [14] and Gordon et al. [15] concluded that CBT/BT with the varying combinations of methods does have efficacy in reducing dental fear, and that CBT/BT can be delivered by staff even with varying educational levels [15]. However, in many studies, follow-up times have been rather short (1 month to 5 weeks) or data on the length of the follow-up period can be missing [15]. Oral conscious or inhalation sedation and hypnosis can be used to support CBT/BT. To help a fearful patient cope with dental treatment, abovementioned techniques have been administered since the year 2000 chair-side in the Clinic for Fearful Dental Patients (CFDP) according to individual needs to provide the patient with coping skills for dental situations. The CFDP functions within primary health care, and the study population was patients of the Municipality of Oulu, Finland, during entire follow-up time.

The aim of the present, retrospective, practice-based study was to analyze the outcome of individually designed chair-side dental fear treatment (CBT/BT) with one to six years' follow-up times among patients referred to the Clinic for Fearful Dental Patients (CFDP) in the primary oral health care in the city of Oulu, Finland.

## 2. Materials and Methods

### 2.1. Study Population

The study population comprised patients (*n* = 163), who had been referred to the CFDP for the treatment in the city of Oulu, Finland, due to dental fear or lack of cooperation during the period 2000–2006. The study population included healthy as well as medically compromised and disabled individuals who however could cope with the fear treatment. Patients were referred to CFDP from the primary oral health care clinics. A criterion was that the referring dentist had tried treating the patient himself before referral. Obligation was also that the referring dentists reported the cause for referral (the fear of dental care in general, the fear of specific procedures or local anesthesia, lack of cooperation, gagging, or something else). In patient records, both a relevant cause (ICD-10) for referral as well as information on the later success of their dental treatment had to be found.

All Finnish children and adolescents are customers in the primary oral health care, and treatment is free of charge for those under 18 years. All participants here were customers of the City of Oulu, Oral and dental health services; therefore, data were reliable and easily available. Data were collected in the fall 2006 with the permission of the register keeper, the City of Oulu, Finland, manually both from paper and electronical patient records (the records before 2003 had been filled manually). Afterwards, the collected information comprised an electronic SPSS database.

### 2.2. Methods

None of the referred patients refused to be treated in the CFDP. Patients were treated in the CFDP by three experienced clinical practitioners, specially trained in treating patients with dental fear (TK, VA, and SK). Dental fear was recognized and diagnosed by interviewing in the CFDP at baseline according to criteria set by Milgrom et al. [16]. For all patients, individual coping techniques for dental anxiety-arousing situations were designed and administered chair-side according to the individual needs. Behavioral, suitable approach (BT) (desensitization, relaxation, distraction, positive reinforcement, and guided imagery), sometimes combined with cognitive restructuring (CBT), i.e., to reduce the sense of catastrophizing/losing control, were the main tools for treating dental fear. The cornerstones were increasing the patient's sense of control, enhancing to trust as well mutual interaction. Pain control was the important part of protocol, i.e., to reduce pain in administering local anesthetics, and the computer aided system for the local anesthesia was used (Wand® Dental, Inc. Livingston, NJ, USA). Additionally, either oral conscious sedation (midazolam, max dose 7.5 mg, for children 0.2–0.5 mg/kg) or nitrous oxide/oxygen inhalation sedation (N_2_O; individually determined concentration 20–50%) were administered, if conscious sedation was considered necessary. In such cases, patients' oxygen saturation was monitored. Some patients were also referred to the dental general anesthesia (DGA) in the University Hospital of Oulu, Finland. While treating dental fear, all dental treatments were accomplished in CFDP, too. The cases and treatment protocols were frequently discussed by the treatment team (TK, VA, and SK).

The outcome for the success of treatment of dental fear was registered as successful/not successful. Dental fear treatment was considered successful if dental treatment in the primary oral health care clinic following the treatment in the CFDP was carried out without any difficulties registered in the patient records: without the need for sedation and without any notices about dental fear or lack of cooperation. Second referral to the CFDP was also recorded (yes/no), and it indicated unsuccessful treatment. The following information was also collected from the patient files: age at the time of referral (year), gender (m/f), cause for the referral (ICD-10) including general, mental, and dental health problems, the starting and ending dates of treatment, and number of visits (*n*) in the CFDP conscious sedation used (pharmaceutical sedation/nitrous oxide; yes/no), and use of DGA (yes/no). Treatment given in the CFDP was registered as restorations, extractions, and orthodontic treatment (yes/no).

For additional information, all participants were sent a questionnaire in the summer 2006 concerning the success of the treatment of dental fear. The participants/parents gave an overall score for the treatment (4, fail, to 10, excellent). The perception of the patient/guardian on treatment in CFDP versus their expectation was asked (as expected/better as expected/worse than expected/not at all) as well as their perception on treatment in primary oral health care afterwards (very well/fairly well/poorly/not at all). The patient-reported success was dichotomized as very or reasonably good = successful and poor or nonexistent = not successful.

### 2.3. Statistics

The length of the monitoring period was calculated from the end date of the treatment in the CFDP. The study population was categorized into three groups according to the age: 2–6, 7–12, and ≥13 years as treatment strategies vary according to the age. The comparisons of the outcome of the treatment in different treatment categories were analyzed using cross tabulation. Statistical significance at *p* < 0.05 level was assessed using the chi-square test. All analyses were executed with the SPSS (version 24.0, SPSS, Inc., Chicago, Il, USA).

### 2.4. Ethics

Data were collected with the permission of the register keeper, Oral Health Section, the City of Oulu, Finland. For this kind of practice-based retrospective follow-up study, neither statement nor permission from the ethical board is required when analyses are carried out without any identification.

## 3. Results

In the study population (*n* = 163), 45% were 2–6-year-olds, 40% were 7–12-year-olds, and 15% were 13-year-olds or older. More than half were males (58.0%), and their proportion was the biggest in the age group ≥ 13 years (Table 1). Thirty participants (*n* = 30, 18.4%) were either medically or mentally compromised; 17 of them had disabilities. The most common cause for referral reported by the referring dentist was the fear of dental treatment in general (Table 2). During the treatment period in the CFDP, restorative treatment was the most common type of treatment (87.7%); other treatments were extractions (4.9%) and orthodontic treatment (1.2%). Length of the observation period from the last visit in the CFPD until data collection was on average 2 y 3 m (SD 1 y 5 m).

**Figure 1:**
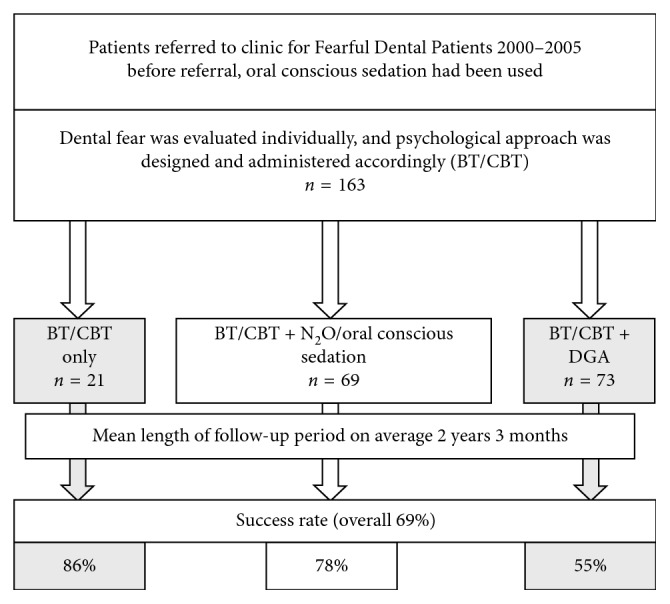
Flowchart describing the protocol and outcome of dental fear treatment in primary health care.

The overall success rate after the treatment period in the CFDP was 68.7% (Figure 1). The outcome was not significantly associated with gender (females 69.6%, males 68.1%). However, the success seemed to be better for those treated at the age of 12 years or younger compared with those treated after they had turned 13. On the contrary, 81.3% of those with disabilities were treated under DGA as a part of treatment in the CFDP, and later on, almost half of them (43.8%) could be treated successfully in normal setting. The overall success rate of the ones without disabilities was 69.6% (*p*=0.038).

BT/CBT was used for all participants and as an only method for 12.9%; the proportion of those with the successful outcome was the biggest in the BT/CBT only group (Table 3). Oral conscious and nitrous oxide sedation and DGA were used if needed. Oral conscious sedation and DGA were most often used in the youngest age group, whereas nitrous oxide was most commonly used among the older ones. Oral conscious sedation (*p*=0.300) or nitrous oxide (*p*=0.585) were not associated with the success of later dental care. The success in accomplishing dental treatment after treating dental fear was significantly better for those who were not treated under DGA (81%) compared with those who were (55%, *p* < 0.001) (Table 3). Later success in dental care among those without disabilities was somewhat better than among those with disabilities (Table 3).

On average, the length of the treatment period was 10.7 months (SD 9.80 months); the outcome success was not associated with it. Participants with the successful outcome had on average five visits (SD 4.3) and the rest four visits (SD 2.3) (n.s.). Of the original study group, 7% were referred again for treatment in the CFDP.

Only 37 (23%) participants responded to the questionnaire (Table 4). The treatment in the CFDP was, in general, reported having been successful and received a mean score of 8.3/10 (SD 1.39). The reported outcome in the primary dental health care after the treatment in the CFDP was similar with the one discovered from the patient records (67.7% reporting very or fairly good success). In the free commentary, the patients or care givers expressed appreciation especially for sufficient time for treatment and an individual approach.

## 4. Discussion

With the monitoring period of in average two years, implementing the theory of dental fear treatment in clinical practice appears useful. Treating dental fear by the chair-side psychological approach and simultaneously accomplishing dental treatment was successful for over two-thirds of patients in this practice-base retrospective study based on the patient files of those treated in the dental fear clinic (CFDP). The outcome was considered successful when any dental treatment afterwards could be given without any notices of fear or lack of cooperation or use of sedatives in patient files. Usefulness of BT/CBT has also been reported by Berge et al. [17], and their findings are in line with the recent study.

BT/CBT alone had the highest association with the positive outcome of treatment in CFDP followed by BT/CBT combined with oral conscious or inhalation sedation. These findings are in line with those of Wide Boman et al. [14] and Gordon et al. [15]. Gomes et al. preferred a cognitive approach over behavioral management techniques [18]. The outcome in most studies is reduction in dental fear and change in oral health-related quality of life according to formal questionnaires [15, 19]. The outcome here was practical, measuring how well the patients could cope in dental situations when they had been provided with individual coping tools. Our results support the statement of Savanheimo et al. [7] that DGA alone does not enable individual return to normative dental care. The findings also emphasize the importance of the individual psychological approach in evaluating dental fear and treating fearful dental patients as has been reported by Wannemueller et al. [8] and Vika et al. [20].

Sample size was enough to detect differences, but in grouped analyses, larger sample size would have been valuable. No one was excluded from the study population regarding their disabilities and medical or dental problems. Berge et al. [17] also with a good outcome of dental fear treatment excluded mentally and intellectually disabled and drug and alcohol abusers. Eight in ten of those with disabilities were treated under DGA here. Their future success rate was lower than that of the healthy ones however comprising almost half of this challenging group. If we had excluded the disabled, the outcome would have been better; however, even those with disabilities also seem to benefit from the chair-side dental fear treatment approach.

The treatment need among the study group was evident, all had dental treatment need of some kind, and almost nine in ten needed restorative care. Nicolas et al. [21] showed the existence of dental fear among those with even one decayed tooth. Patients with the best dental health and lowest treatment needs have been shown to have the best response for fear treatment [22]. Because most participants had treatment need and yet the outcome was good for almost 70% and less than 10% returned to the CFDP the second time, the present approach seems worth more studies.

The participants whose dental fear was treated in early years of life had a better outcome than those who were only treated after 13 years of age. In all likelihood, older participants referred to the CFDP had more load of direct and indirect causes for dental fear [23]. Our results indicate that an early intervention to dental fear is most efficient.

More males than females were referred to the clinic. The cause for referring males more often to dental fear treatment than females is may be due to their actually poorer oral health status. The outcome of the dental fear treatment itself was equally good for both genders. The school health surveys from Finland show that boys are lazier in tooth brushing and have dentally more harmful dietary habits than girls [24]. Harmful habits cause a risk for oral diseases like dental caries, which require dental treatment, which again maybe painful and consequently may cause dental fear [21]. Boys may also be more challenging to handle in dental situations and therefore get more easily referral than girls, yet this was not studied here. Chapman and Kirby-Turner [25] have stated that uncooperative behavior might lead to referral to specialized care. Lack of cooperation and behavior management problems may falsely be considered as a sign of dental fear and are definitely more easily recognized than fear.

On average, treatment in the CFDP lasted less than a year and the mean number of visits was about five, which was considerably low, when dental procedures were carried out simultaneously with the chair-side dental fear treatment. This protocol may be one reason for the good outcome—all the challenging procedures for the patient were accomplished in the CFDP before returning to primary health care. Tools were also given to cope with routine procedures in future both to the patient and the dentist. In addition to individual benefits, success in treating patients with fear may also be economically favorable for the organization in a long run, due to reduction in missed appointments and appointments without any achieved procedures [26].

The data of the present practice-based study comprise patient records. In Finland, all citizens are entitled to dental care subsidized by the society, those under 18 even to free dental care. Patient records in municipalities are readily available for research purposes with the permission of the register holder and have been found to be reliable [27]. The dentists are obliged to record all visits and findings. This is an advantage in this study. The study sample was limited, yet big enough to enable comparing the outcome of the dental fear treatment in age groups and between genders. The study sample size is similar to other respective studies [17, 28].

Only a limited proportion of the participants agreed to fill the VAS-, MDAS-, and GFS forms to describe in detail their dental fear. Sometimes, they were not even given to patients for a variety of reasons. These forms would have been most valuable in describing the study group and following the outcome of the dental fear treatment. This is a limitation, even if Milgrom et al. [16], is not restrictive in this sense. The referring dentists, however, gave a cause for referral, which practically in all cases was lack of cooperation/dental fear.

All participants were sent a questionnaire for a self-reported evaluation of the treatment. The respondents' number was 37 (13%) (either patients or care givers). In a recent article by Rodd et al. the response rate to a questionnaire was about 50%, yet the outcome was good to a self-help CBT [29]. Some parents of the children with learning disability phoned afterwards telling that they found answering difficult and were agreed not to participate in the survey. Indeed, it is a limitation that the profile of those responding to the questionnaire could not be compared with the nonresponders because IDs were not asked in the questionnaire. Despite the low response rate, the outcome was in concordance with the findings based on patient records. Valuable information was also received in free commentary: sufficient time for treatment, an individual approach, and a good attitude of the dental personnel were factors appreciated by the patients. The similar conclusion was also suggested by Morhed Hultvall et al. [30].

The simple chair-side protocol practiced in the CFDP allows patients to be treated successfully in primary dental health care providing the patients and dentists with tools for coping in dental situations later on. This is beneficial for the individual and for the organization and can improve individual's oral health-related quality of life [9]. Cost-efficacy is evident but needs investigation. This study encourages dentists in primary health care to learn the basics of dental fear treatment to use them with sufficient time when treating fearful patients and see the value of such work.

## Figures and Tables

**Table 1:** Age-specific distribution (*n* (%)) by gender, treatments given in CFDP as well as outcome of the fear treatment afterwards among the participants referred to CFDP.

	All	2–6 yrs	7–12 yrs	≥13 yrs	*p*
Participants	163 (100)	73 (45)	65 (40)	25 (15)	
Females	69 (42)	33 (45)	32 (49)	4 (16)	0.013
Persons with disabilities	18 (11)	2 (3)	7 (11)	9 (36)	<0.001
BT/CBT only	21 (13)	8 (11)	11 (17)	2 (8)	0.424
Oral sedation	94 (58)	52 (71)	37 (57)	5 (20)	<0.001
N_2_O	41 (25)	11 (15)	20 (31)	10 (40)	0.019
General anesthesia	73 (45)	33 (45)	25 (38)	15 (60)	0.183
Successful outcome	112 (69)	53 (73)	47 (72)	12 (48)	0.053

**Table 2:** Causes reported by the referring dentist at the time of the referral of the patient to the CFDP.

Indication for referral to CFDP	*n*	%
Fear of dental treatment in general	91	67
Fear of needles	15	11
Fear of procedures	12	9
Immaturity for dental treatment	12	9
Generalized fear	2	2
Gagging	3	2
Total	163	100

**Table 3:** Successful outcome of dental fear treatment (*n* (success %)) according to age, disabilities, and the treatment type: only BT/CBT or combined with oral conscious sedation and/or N_2_O or DGA.

Treatment type	Age groups			Disabilities		All
	2–6 yrs	7–12 yrs	≥13 yrs	Yes	No	
*n*	73	65	25	18	145	163
BT/CBT only	8 (100)	11 (82)	2 (50)	1 (0)	20 (90)	21 (86)
BT/CBT + oral conscious sedation and/or N_2_O	32 (78)	29 (83)	8 (63)	3 (67)	66 (79)	69 (78)
BT/CBT + DGA	33 (61)	25 (56)	15 (40)	14 (43)	59 (58)	73 (55)
*p*	0.052	0.067	0.588	0.493	0.005	0.002

**Table 4:** Self-reported perceptions of treatment in the CFDP and outcome in the basic health care after the treatment (%).

Self-reported outcome	Scale	2–6 yrs (%), *n* = 15	7–12 yrs (%), *n* = 17	≥13 yrs (%), *n* = 5
Perception of treatment	As expected	47	47	80
	Better than expected	40	35	20
	Worse than expected	13	12	0
	Not at all	0	16	0

Outcome in the basic health care	Very good	33	36	20
	Reasonably good	33	43	20
	Poor	25	7	20
	Nonexistent	9	14	40

## Data Availability

The data used to support the findings of this study are available from the corresponding author upon request.
